# Post-Hypoxic Cells Promote Metastatic Recurrence after Chemotherapy Treatment in TNBC

**DOI:** 10.3390/cancers13215509

**Published:** 2021-11-02

**Authors:** Inês Godet, Mahelet Mamo, Andrea Thurnheer, D. Marc Rosen, Daniele M. Gilkes

**Affiliations:** 1 Department of Oncology, The Sidney Kimmel Comprehensive Cancer Center, The Johns Hopkins University School of Medicine, Baltimore, MD 21231, USA; ines.godet@jhu.edu (I.G.); mahelet.mamo@live.longwood.edu (M.M.); Rosenma@jhmi.edu (D.M.R.); 2 Department of Chemical and Biomolecular Engineering, The Johns Hopkins University, Baltimore, MD 21218, USA; athurnh1@jhu.edu; 3 Johns Hopkins Institute for NanoBioTechnology, The Johns Hopkins University, Baltimore, MD 21218, USA; 4 Cellular and Molecular Medicine Program, The Johns Hopkins University School of Medicine, Baltimore, MD 21231, USA

**Keywords:** intratumoral hypoxia, breast cancer metastasis, recurrence

## Abstract

**Simple Summary:**

Intratumoral hypoxia is a negative prognostic factor in breast cancer progression and recurrence. By implementing a hypoxia fate-mapping system, we followed cells that experience intratumoral hypoxia in vivo and determined that these cells have an increased ability to metastasize compared to cells that were never exposed to hypoxia. In this work, we investigate whether cells that experienced intratumoral hypoxia are also resistant to chemotherapy. By utilizing both in vivo and ex vivo models, we conclude that metastatic cells found in the lung and liver, that were exposed to hypoxia in the primary tumor, are less sensitive to doxorubicin and paclitaxel and drive recurrence after treatment. Our studies also suggest that chemoresistance is associated with a cancer stem cell-like phenotype that is maintained in post-hypoxic cells.

**Abstract:**

Hypoxia occurs in 90% of solid tumors and is associated with treatment failure, relapse, and mortality. HIF-1α signaling promotes resistance to chemotherapy in cancer cell lines and murine models via multiple mechanisms including the enrichment of breast cancer stem cells (BCSCs). In this work, we utilize a hypoxia fate-mapping system to determine whether triple-negative breast cancer (TNBC) cells that experience hypoxia in the primary tumor are resistant to chemotherapy at sites of metastasis. Using two orthotopic mouse models of TNBC, we demonstrate that cells that experience intratumoral hypoxia and metastasize to the lung and liver have decreased sensitivity to doxorubicin and paclitaxel but not cisplatin or 5-FU. Resistance to therapy leads to metastatic recurrence caused by post-hypoxic cells. We further determined that the post-hypoxic cells that metastasize are enriched in pathways related to cancer stem cell gene expression. Overall, our results show that even when hypoxic cancer cells are reoxygenated in the bloodstream they retain a hypoxia-induced cancer stem cell-like phenotype that persists and promotes resistance and eventually recurrence.

## 1. Introduction

One in every eight women will develop breast cancer in their lifetime, and it has been projected that breast cancer will cause over 42,000 deaths this year [1]. The estimated 5-year survival rate for patients with localized breast cancer is 99%; however, this number dramatically drops to 27% when distant metastasis is detected [1]. Breast cancer metastasis occurs when cancer cells spread from the primary tumor through the body, typically to the lung, liver, bone, or brain [2]. It is estimated that 30% of patients with advanced breast cancer will develop distant metastasis [3], which is the main cause of treatment failure and mortality, leading to 90% of cancer-related deaths [4]. Even in patients with tumors that are responsive to radiotherapy or chemotherapy, metastasis is frequently a cause of treatment failure [5]. Metastatic tumors are anatomically less accessible, which makes conventional treatment options such as surgery or radiotherapy extremely challenging [6]. Moreover, metastatic lesions may arise from the clonal evolution of selected aggressive cancer cells that overcome a series of obstacles to thrive at a distant site [7]. In breast cancer, these cells are often pre-exposed to systemic therapy used to treat the primary tumor or to prevent recurrence [8]. Together, these circumstances make metastatic disease nearly incurable [9].

The standard of care for triple-negative metastatic breast cancer (TNMBC) is typically chemotherapy. The main cytotoxic agents used against TNMBC primarily target cell division. For instance, cisplatin binds to the N7 reactive center on purine residues, causing DNA damage in cancer cells, blocking cell division, and resulting in apoptotic cell death [10]. Doxorubicin can act on cancer cells by two main mechanisms: (1) intercalation into DNA and disruption of topoisomerase-II-mediated DNA repair and (2) generation of free radicals that damage cellular membranes, DNA, and proteins [11]. 5-Fluorouracil (5-FU) is converted to fluorodeoxyuridine monophosphate. This forms a stable complex with thymidylate synthase and inhibits deoxythymidine monophosphate (dTMP) production, which is vital for DNA replication and repair, leading to cytotoxicity [12]. Paclitaxel is a microtubule-stabilizing drug that induces mitotic arrest, leading to cell death [13]. The overall contribution of cytotoxic chemotherapy to the 5-year survival rate of adult cancer patients is estimated to be 2.1% in the United States, suggesting that this nontargeted approach has a low success rate [14].

The rapid proliferation of cancer cells along with the lack of efficient vasculature causes regions of hypoxia to develop in 90% of solid tumors [15,16]. Studies on pretreatment oxygenation status of solid tumors revealed that the median partial pressure of oxygen (PO_2_) in breast tumors is 10 mmHg (1.3% O_2_) in contrast to 65 mmHg (8% O_2_) in normal breast tissue [17]. Hypoxic PO_2_ values (<10 mmHg) have been correlated with increased risk of metastasis and mortality [18,19,20]. The most well-characterized response to oxygen deprivation is via the stabilization of the hypoxia-inducible subunits (HIF-1α and HIF-2α) [21]. Stabilized HIF-1/2α subunits heterodimerize with HIF-1β to form the HIF-1/2 complexes that recognize a 5′-ACGTG-3′ enhancer sequence and transcriptionally regulate more than a thousand genes [22,23]. Hypoxia-induced HIF-1/2 activation has been implicated in multiple mechanisms of chemoresistance, including ABC transporter activity, autophagy, DNA repair activation, enrichment in cancer stem cell-like properties, and repression of apoptosis and senescence [24].

We previously developed a hypoxia fate-mapping system that triggers a permanent fluorescent switch from DsRed to GFP when cells experience intratumoral hypoxia leading to increased HIF-1/2 expression. Our studies using breast cancer murine models revealed that GFP+ cells are 5 times more likely to successfully establish a metastatic lung lesion by adopting a ROS-resistant phenotype [25]. Given that hypoxia has been widely associated with chemoresistance, we sought to investigate whether these potent metastatic cells (hereinafter called post-hypoxic cells) also have decreased sensitivity to standard-of-care chemotherapeutic agents. In this work, we use both ex vivo and in vivo approaches to show that post-hypoxic cells (GFP+) are more resistant to doxorubicin and paclitaxel, but not cisplatin or 5-FU, and are more likely to contribute to recurrence following treatment.

## 2. Materials and Methods

### 2.1. Cell Culture

Mycoplasma-free breast cancer cell lines MDA-MB-231 and 4T1 were obtained from the American Type Culture Collection (ATCC) and maintained in DMEM and RPMI (Sigma-Aldrich, St. Louis, MO, USA), respectively, with 10% FBS (Corning, Corning, NY, USA) and 1% penicillin/streptomycin (Invitrogen, Carlsbad, CA, USA).

### 2.2. Hypoxia Fate-Mapping System

We previously generated and characterized the MDA-MB-231 and 4T1 hypoxia fate-mapping cell lines [25]. The vectors used to generate these cell lines, CMV-loxp-DsRed-loxp-eGFP (#141148) and 4xHRE-MinTK-CRE-ODD (#141147), are available at Addgene.

### 2.3. Tumor Implantation and Surgical Resection

Female 6- to 8-week-old NOD-SCID Gamma (NSG) mice were utilized according to protocols approved by the Johns Hopkins University Animal Care and Use Committee. Mice were anesthetized with 100 mg/kg ketamine and 16 mg/kg xylazine (Vet One) via intraperitoneal injection. MDA-MB-231 (2 × 10^6^) or 4T1 (500) hypoxia fate-mapping cells were injected into the mammary fat pad under the second left nipple. Tumors were resected 22 to 25 days after tumor implantation. Briefly, mice were anesthetized and transferred to a heating pad. After hair removal, the tumor was carefully detached from adjacent skin, removing adjacent loose tissue and visible lymph nodes, and the wound was closed using 9 mm autoclips (Braintree Scientific, Inc.; Braintree, MA, USA). Betadine was applied to the wound and eye desiccation was prevented by using ophthalmic ointment. Resected tumors were divided in half. Half of the tumor was processed to obtain a cell suspension. Tumor tissue was physically and enzymatically (collagenase 2 mg/mL; MilliporeSigma; Burlington, MA, USA) dissociated, passed through a cell strainer (0.7 μM), and washed. Samples were resuspended in FACS buffer (PBS, 1% BSA, 0.5 mM EDTA, 25 μg/mL DNAse) and analyzed using an SH800 (Sony; San Jose, CA, USA) flow cytometer. The other half of the tumor was formalin-fixed, saturated in 30% sucrose (Sigma-Aldrich; St. Louis, MO, USA) at 4 °C overnight, embedded in OCT media (Fisher Scientific; Hampton, NH, USA), frozen in liquid nitrogen, sectioned via a cryotome CM1100 (Leica; Wetzlar, Germany), and mounted onto Superfrost Plus Microscope Slides (Fisher Scientific). Tumor sections were stained with DAPI (1:1000 for 15 min, RT), mounted with antifade solution, and imaged using an Olympus (UPLFLN 4X) objective on a Cytation 5 instrument (BioTek Instruments; Winooski, VT, USA).

### 2.4. Generation of Tumor-Derived Cell Lines

Tumor-derived cells were obtained following the previously described tissue dissociation protocol with physical dissociation, followed by enzymatic digestion (collagenase 2 mg/mL (Sigma-Aldrich) and BSA 2 mg/mL (Gemini Bioproducts; West Sacramento, CA, USA) for 1 h at 37 °C and 160 RPM. The resultant cell suspension was washed with PBS, resuspended in sorting buffer (PBS, 1% BSA, 0.5 mM EDTA, 25 mM HEPES pH 8), and sorted into DsRed+/GFP− and DsRed−/GFP+ populations using an SH800 Cell Sorter (Sony Biotechnology). GFP was detected in the FITC channel, and DsRed was detected in the PE channel. Cells were immediately plated in tissue-culture plates with warm DMEM or RPMI media. Cells were mycoplasma-tested and maintained in culture for at most 5–6 passages.

### 2.5. Ex Vivo Drug Screening

Tumor-derived MDA-MB-231 and 4T1 DsRed+ or GFP+ cells were plated in 96-well plates (5000 cells/well). The following day, fresh media with cisplatin, doxorubicin, 5-FU, or paclitaxel were added. In order to obtain IC_50_ curves, 7 different drug concentrations and a control condition were evaluated for each chemotherapeutic agent. Following 48 h, the media were aspirated and media with 10% Presto Blue were added. Plates were incubated for 2 h and fluorescence was read using a Cytation5 (BioTek Instruments). The cells were then washed with PBS, fixed with 4% PFA, and stained with DAPI. The entire tissue culture well was imaged in the RFP, GFP, and DAPI channels with an Olympus (UPLFLN 4XPh) phase objective 4× in a montage (4 × 3) (BioTek Instruments). Images were linearly stitched, and the DAPI area was quantified by thresholding as a measure of cellular confluence. IC_50_ curves based on fluorescence measured by Presto Blue or cell confluence were obtained using the nonlinear fit log(inhibitor) vs. response model available in the GraphPad Prism software. GFP+ tumor-derived cells only retained a resistant phenotype against doxorubicin and paclitaxel for 5–6 passages.

### 2.6. Ex Vivo Colony Formation Assay

Tumor-derived MDA-MB-231 DsRed+ or GFP+ cells were plated in 96-well plates (5000 cells/well) and treated with the 10 nM of paclitaxel for 48 h, followed by a 30-day recovery period to allow resistant colonies to form. Media were refreshed twice a week, and whole wells were imaged in the RFP and GFP channels with an Olympus (UPLFLN 4XPh) phase objective 4× in a montage (4 × 3) (BioTek Instruments). Images were linearly stitched, and colonies were counted using the images acquired on day 20 of the experiment.

### 2.7. Chemotherapeutic Treatment of Mice

Chemotherapy treatment was administered to animals via intravenous injection. Briefly, mice were warmed for 5 min with an overhead heat lamp to dilate the veins, while applying friction to the tail. Each animal was restrained, and the dose of chemotherapy (3 mg/kg cisplatin, 2 mg/kg doxorubicin, 20 mg/kg 5-FU, or 16 mg/kg paclitaxel) was injected in appropriate vehicle solution using a 26 G needle. The control group was treated with vehicle solution only.

### 2.8. Analysis of Metastatic Progression

At the end point of the experiment, animals were sacrificed to assess metastatic burden. Lungs were inflated with an OCT–PBS solution, excised, and immediately formalin-fixed. Livers were excised and fixed. Whole mount fluorescent imaging of lungs and livers was conducted using an Olympus (UPLFLN 1.25XPh) phase objective 1.25× (BioTek Instruments) in the RFP and GFP channels. Images were linearly stitched. The quantification of the metastatic burden was performed using flow cytometry analysis. Briefly, lungs and livers were chopped and digested in 2 mg/mL collagenase (Sigma-Aldrich) and 2 mg/mL BSA (Gemini Bioproducts) for 1 h at 37 °C at 160 RPM. After passing through a 0.70 μm strainer, cells were washed with PBS and resuspended in FACS buffer. GFP and DsRed were detected in the FITC and PE channels, respectively, using an SH800 (Sony Biotechnology). Data were analyzed using the FlowJo V10 software (Tree Star Inc., Ashland, OR, USA).

### 2.9. Computational Analysis

RNA sequencing was previously performed on DsRed+ and GFP+ cells sorted from tumors and lungs of mice [25]. Gene set enrichment analysis (GSEA) [26] was conducted to determine enrichment in DsRed or GFP cells derived from tumors or lungs against gene sets associated with chemoresistance and resistance to cisplatin, doxorubicin, or 5-FU available in the GSEA database and a paclitaxel-resistance gene signature compiled as described in Table S1. Survival curves were plotted using the Kaplan–Meier Plotter (https://kmplot.com/analysis/; accessed on 26 April 2021) online tool using mRNA gene chip data from breast cancer patients [27]. ROC analysis was conducted using the ROC Plotter (http://www.rocplot.org/; accessed on 9 April 2021) online tool [28].

### 2.10. Mammosphere Formation Assay

Freshly resected MDA-MB-231 tumors were subjected to physical dissociation followed by enzymatic digestion (collagenase 2 mg/mL (Sigma-Aldrich), BSA 2 mg/mL (Gemini Bioproducts)) for 1 h at 37 °C at 160 RPM. Cells were then passed through a cell strainer (0.7 μM), and 10,000 cells/well were transferred to a 6-well plate previously coated with polyHEMA (12 g/L in 95% EtOH) and air-dried for 48 h with 2 mL of mammosphere formation media per well (MammoCult basal medium (human) with 4 ug/mL heparin, and 0.48 ug/mL hydrocortisone) (STEMCELL Technologies; Vancouver, BC, Canada).

### 2.11. Statistical Analysis

All data are presented as mean ± SEM, and statistical analysis was performed using GraphPad Prism 9. Differences in metastatic burden were analyzed via ordinary one-way ANOVA with Dunnett (recommended) multiple comparison test or paired one-tailed Student’s *t*-test when comparing DsRed versus GFP. A comparison of DsRed+ versus GFP+ in matched samples such as the mammosphere assay was performed via paired two-tailed Student’s *t*-test. Significance levels are reported by displaying exact *p*-values.

## 3. Results

### 3.1. Post-Hypoxic Cells Are Less Sensitive to Doxorubicin and Paclitaxel but Not 5-FU or Cisplatin Treatment Ex Vivo

**Figure 1:**
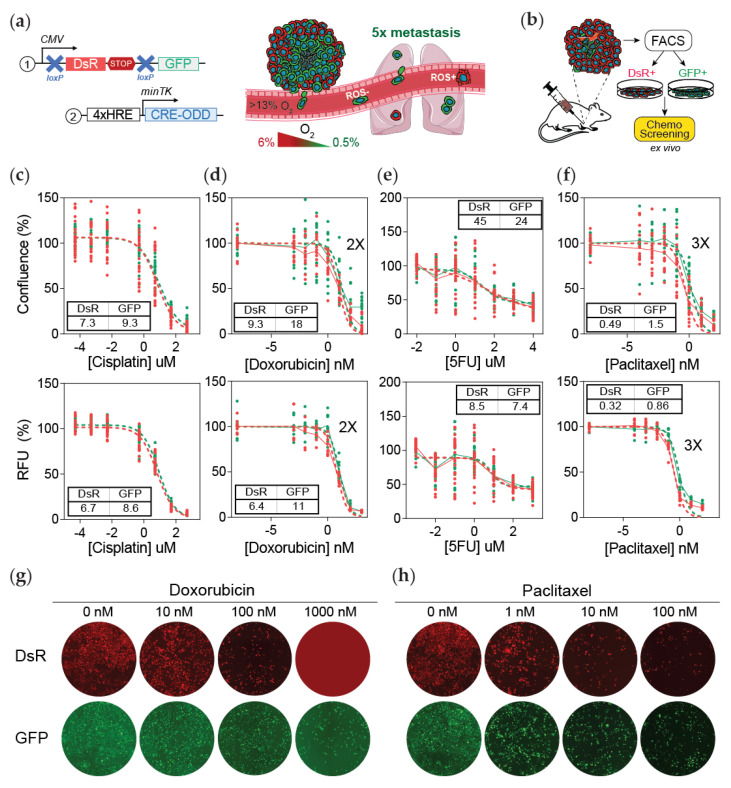
Post-hypoxic cells are less sensitive to doxorubicin and paclitaxel but not 5-FU or cisplatin treatment ex vivo. (**a**) Lentiviral vectors were delivered to MDA-MB-231 cells to generate a hypoxia fate-mapping system (left). A cartoon depicting that cells that experienced intratumoral hypoxia (GFP+) have a higher probability of forming lung metastases (right). (**b**) Schematic of the experimental set-up for (**c**–**h**). Tumors that formed from the orthotopic injection of MDA-MB-231 hypoxia fate-mapping cells in NSG mice were sorted into DsRed+ or GFP+ populations and then cultured in vitro to determine the IC_50_ of cisplatin (**c**), doxorubicin (**d**), 5-fluorouracil (**e**), or paclitaxel (**f**) after 48 h of treatment. IC_50_ curves were generated by assessing cell viability using the area of the cell culture well covered by nuclear DAPI staining (top) or using a Presto Blue assay (bottom) (N = 3, *n* = 3); GFP versus DsRed. RFU = relative fluorescence units. (**g**,**h**) Fluorescent images of tumor-derived DsRed+ and GFP+ sorted cells treated with doxorubicin (**g**) or paclitaxel (**h**) using increasing drug concentrations as indicated. Images are quantified in Figure S1f.

We recently developed a dual-vector lentiviral system that when expressed in cells causes a permanent fluorescent switch from DsRed to GFP expression under hypoxia. The first vector contains a constitutively active promoter that drives the expression of DsRed. The DsRed gene is flanked by loxp sites and is localized upstream of the GFP gene. The second vector is activated by HIF binding to the hypoxia-responsive elements (HREs) located upstream of a minimal promoter that causes the expression of an altered Cre protein that contains an oxygen-dependent domain (ODD) (Figure 1a). When a cell experiences hypoxia, Cre accumulation drives the cleavage of DsRed and consequently the permanent expression of GFP. By incorporating this system in an MDA-MB-231 orthotopic mouse model of breast cancer metastasis, we previously reported that post-hypoxic cancer cells (GFP+) have a survival advantage in the bloodstream leading to a 5-fold increase in their ability to metastasize to the lung (Figure 1a) [25].

Intratumoral hypoxia has been associated with multiple mechanisms of chemoresistance [24]. We previously performed an RNA sequencing analysis to characterize DsRed+ and GFP+ cancer cells derived from mouse tumor and lung metastasis. Using gene set enrichment analysis (GSEA) [26], we determined that GFP+ cells were enriched in a gene signature associated with chemoresistance [29] (Figure S1a–c). To further explore the impact of hypoxia in resistance, we assessed the effect of four standard-of-care chemotherapies used to treat patients with breast cancer utilizing an ex vivo approach (Figure 1b). DsRed+ and GFP+ MDA-MB-231 cells were sorted from orthotopic tumors harvested 25 days after cancer cell inoculation. After expansion, the IC_50_ for cisplatin, doxorubicin, 5-fluorouracil, and paclitaxel was determined by measuring fluorescence (RFU) using a Presto Blue proliferation assay, as well as by determining the percentage of cell confluence by analyzing the percentage of DAPI staining. The results showed that cells that experienced intratumoral hypoxia (GFP+) have a 2-fold higher IC_50_ for doxorubicin and 3-fold higher IC_50_ for paclitaxel, whereas the IC_50_ values for cisplatin or 5-FU treatments did not change (Figure 1c–h and Figure S1d–f). GFP+ cells sorted from 4T1 orthotopic tumors also displayed decreased sensitivity compared to DsRed+ cells, particularly to paclitaxel treatment (Figure S2).

### 3.2. Post-Hypoxic Cells Are Less Sensitive to Doxorubicin and Paclitaxel but Not Cisplatin or 5-FU Treatment In Vivo

**Figure 2:**
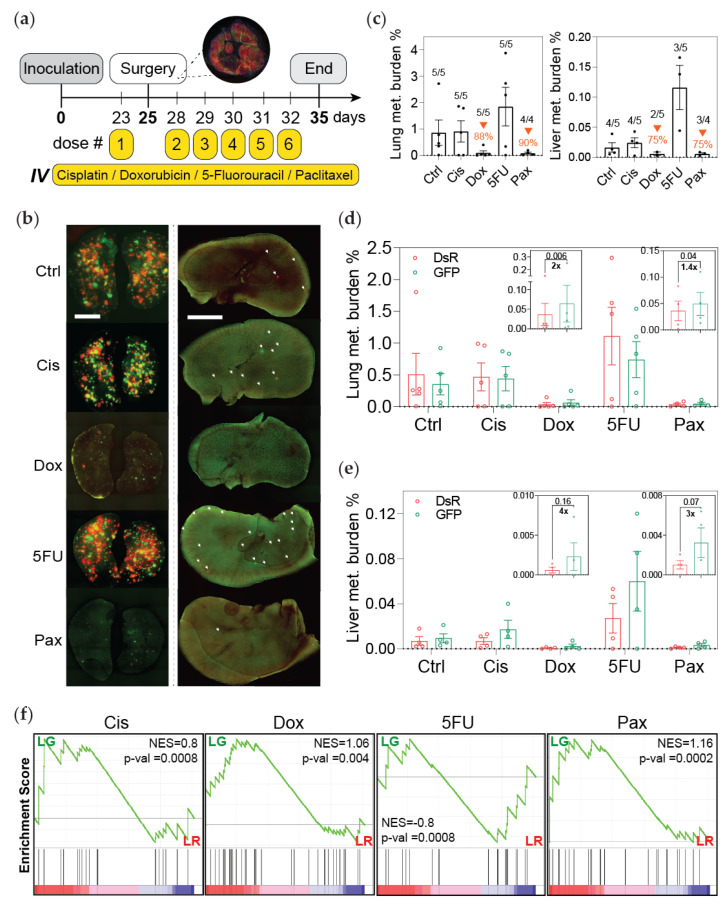
Post-hypoxic cells are less sensitive to doxorubicin and paclitaxel but not cisplatin or 5-FU treatment in vivo. (**a**) A schematic depicting the experimental set-up. Primary tumors were surgically removed 25 days post implantation following a single neoadjuvant dose of chemotherapy (Ctrl = control; Cis = cisplatin; Dox = doxorubicin; 5FU = 5-fluorouracil; Pax = paclitaxel). Three days after surgery, mice were treated with daily doses of chemotherapy (dose #4 of paclitaxel was suspended). Mice were sacrificed to assess metastatic burden 3 days following the last dose. (**b**) Whole mount fluorescent image of lung (left) and liver (right) from one representative mouse per treatment group. (**c**) Percentage of fluorescent (DsRed+ and GFP+) cells measured by flow cytometry analysis (orange arrows indicate decreased metastatic burden compared to the control group). The ratio of mice with detectable metastasis over the total number in the treatment group is displayed for each condition. (**d**,**e**) The percentage of DsRed+ or GFP+ cells in the lung (**d**) and liver (**e**) as measured by flow cytometry (*n* = 4–5); *p*-value is displayed to compare GFP versus DsRed (one-tailed paired *t*-test). Insets show a shorter *y*-axis scale for doxorubicin and paclitaxel treatments. (**f**) Gene set enrichment analysis (GSEA) of gene sets associated with resistance to cisplatin, doxorubicin, 5-FU, and paclitaxel in GFP+ (LG) versus DsRed+ (LR) metastatic cancer cells isolated from the lung. Normalized enrichment score (NES) and *p*-value (*p*-val) are displayed.

To determine whether the sensitivity of GFP+ cells to chemotherapy was also similar in vivo, hypoxia fate-mapping MDA-MB-231 cells were implanted into the mammary fat pad of NSG mice. Tumors were resected 25 days later following a neoadjuvant dose of chemotherapy. We previously demonstrated [25] and confirmed here that at the time of tumor removal about 20% of the cancer cells in the primary tumor had experienced intratumoral hypoxia and were GFP+ (<1% O_2_) (Figure S3a). Five additional doses of chemotherapy were administered daily after surgery, and the mouse weight was monitored over the course of treatment (Figure 2a and Figure S3b,c). Treatment with cisplatin or 5-FU did not reduce metastatic burden in this model, while doxorubicin and paclitaxel reduced or prevented the outgrowth of lung and liver metastasis by 88–90% and 75%, respectively (Figure 2b,c). However, mice treated with doxorubicin or paclitaxel had almost twice the fraction of GFP+ compared to DsRed+ cells in the lung and 3 to 4 times more GFP+ compared to DsRed+ cells in the liver at the endpoint of the experiment (Figure 2d,e). Taken together the data demonstrated that there are 8 and 14 times more GFP+ than DsRed+ cells present in the metastatic lung and liver lesions, respectively, than there were in the primary tumor at the time of removal.

To ensure that the increase in GFP+ cells was not due to paclitaxel promoting HIF-1α activation [30], we performed a control experiment. We injected hypoxia fate-mapping MDA-MB-231 cells into the tail vein of mice in order to directly seed the mouse lung without forming a primary tumor. The mice were treated with five daily doses of paclitaxel. The colonies that formed in the lung were exclusively DsRed+ (Figure S3d). This confirms that paclitaxel-driven HIF1α expression is not sufficient to promote a DsRed-to-GFP switch in our system. Likewise, the DsRed-to-GFP switch occurs in the primary tumor and is maintained when the cells are reoxygenated in the lung, as we previously reported.

By utilizing GSEA analysis of GFP+ compared to DsRed+ cells, we investigated previously published signatures associated with resistance to cisplatin [31], doxorubicin [31], 5-FU [31], and paclitaxel [32,33,34,35] (Table S1). GFP+ cancer cells sorted from either the primary tumor or lung were significantly enriched in genes associated with resistance to doxorubicin, paclitaxel, and cisplatin, but not 5-FU (Figure 2f and Figure S4). Taken together, these data (1) recapitulate the results obtained using the ex vivo screening assay and (2) demonstrate that cells that experience intratumoral hypoxia (GFP+) and then metastasize have decreased sensitivity to doxorubicin and paclitaxel.

### 3.3. Post-Hypoxic Cells Are Less Sensitive to Doxorubicin and Paclitaxel

**Figure 3:**
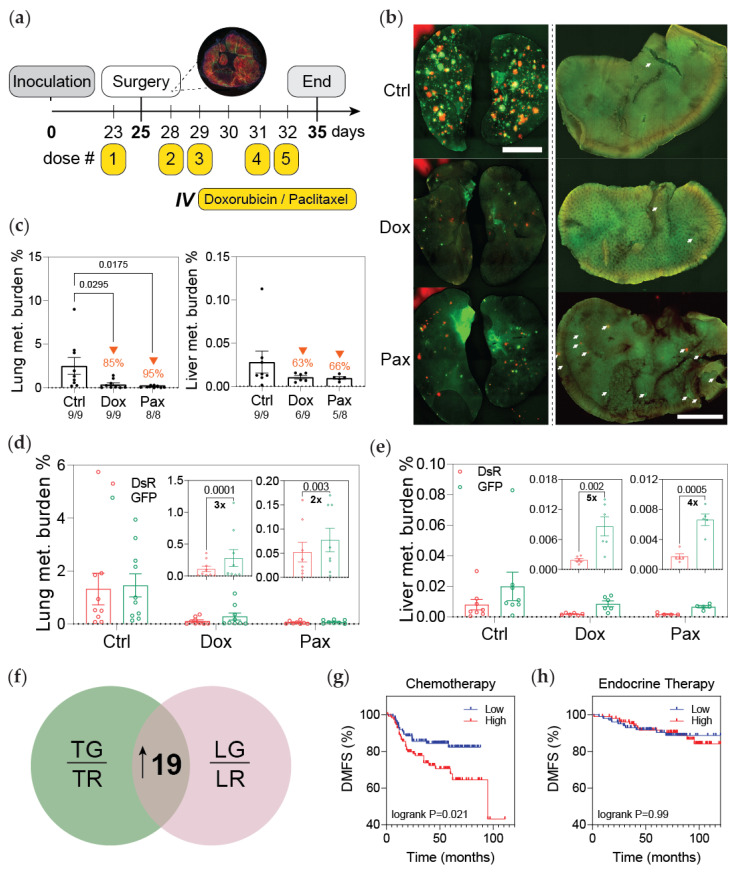
Post-hypoxic cells are less sensitive to doxorubicin and paclitaxel. (**a**) Schematic depicting the experimental set-up: primary tumors were surgically resected 25 days post implantation following one IV dose of neoadjuvant chemotherapy. Three days after surgery, the mice were treated with four doses of chemotherapy; mice were sacrificed 3 days following treatment. (**b**) Whole mount fluorescent image of lung (left) and liver (right) from one representative mouse per treatment group. (**c**) Percentage of fluorescent (DsRed+ and GFP+) cells measured by flow cytometry analysis (orange arrows indicate the decrease in metastatic burden compared to the control group). The ratio of mice with detectable metastasis over the total number in the treatment group is displayed for each condition. (**d**,**e**) The percentage of DsRed+ or GFP+ cells in the lung (**d**) and liver (**e**) as measured by flow cytometry (*n* = 8–9); *p*-value is displayed to compare GFP versus DsRed (one-tailed paired *t*-test). Insets show a reduced *y*-axis scale for doxorubicin and paclitaxel treatments. (**f**) Venn diagram displaying the 19 genes upregulated in both GFP+ (TG) versus DsRed+ (TR) tumor cells and GFP+ (LG) versus DsRed+ (LR) metastatic cells in the lung termed ‘hypoxic memory’ previously reported [25]. (**g**,**h**) Microarray expression data were used to perform Kaplan–Meier analysis of distant metastasis-free survival (DMFS) of breast cancer patients stratified by high or low expression of the ’hypoxic memory’ 19-gene signature and treated with either (**g**) chemotherapy (N = 225) or (**h**) endocrine therapy (N = 205).

Next, we performed a follow-up experiment with a larger cohort of mice using the same treatment regimen consisting of a neoadjuvant dose of chemotherapy, tumor resection, and four consecutive doses of doxorubicin or paclitaxel (Figure 3a–c and Figure S5a–c). The results replicated our initial findings showing a higher fraction of GFP+ versus DsRed+ cancer cells in the lung and liver of mice treated with chemotherapy versus the vehicle control (Figure 3d–e). To validate our findings in a second model of breast cancer metastasis, we performed the same study using 4T1 hypoxia fate-mapping cells. Tumors were resected at day 22 when they were comprised of about 15% GFP+ cells (Figure S5d). Animals were monitored over the course of the experiment (Figure S5e,f). Paclitaxel treatment reduced metastatic lung burden by 45%. The fraction of GFP+ cells in the lung was 2-fold more than DsRed cells (Figure S5g–i). Together, these data confirm that cells that experience intratumoral hypoxia (GFP+) and metastasize to distant organs are less sensitive to paclitaxel.

In our previous work, we identified a 19-gene signature upregulated in GFP+ cells derived from both tumor and lung metastases that we termed ‘hypoxic memory’ genes [25] (Figure 3f). By utilizing this signature, we compared the distant metastasis-free survival (DMFS) of patients treated with chemotherapy (N = 225) or endocrine therapy (N = 205) [27]. The patients were stratified as either having high or low expression of the 19-gene signature using median expression as the cutoff. Patients treated with chemotherapy that had high expression of the 19-gene signature had a worse outcome (Figure 3g). On the other hand, the expression of this signature was not prognostic for patients treated with endocrine therapy (Figure 3h). These observations suggest that the post-hypoxic phenotype could be associated with reduced sensitivity to chemotherapy.

### 3.4. Post-Hypoxic Cells Are More Likely to Contribute to Metastatic Recurrence after Treatment

**Figure 4:**
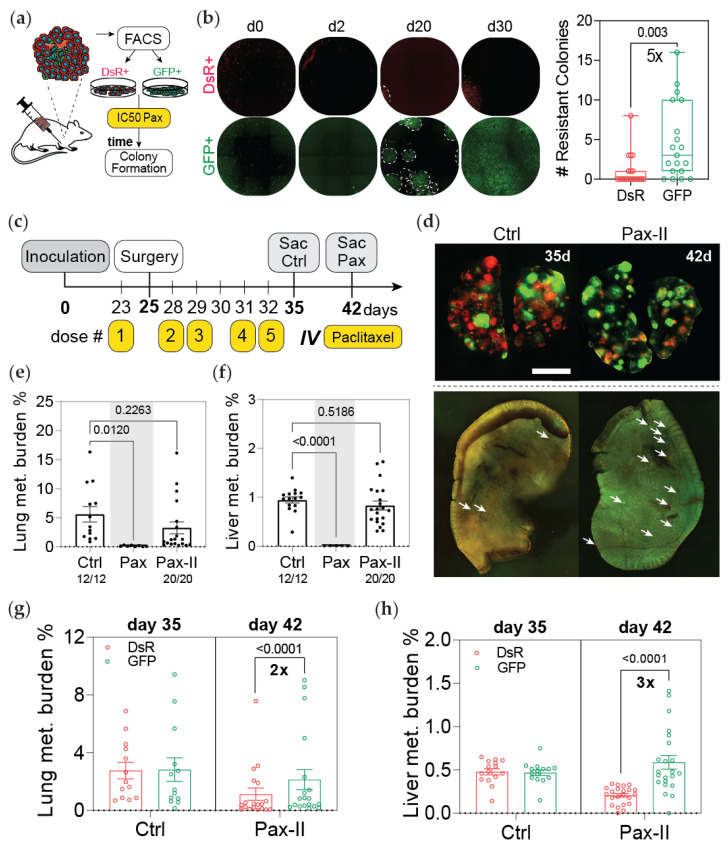
Post-hypoxic cells are more likely to contribute to metastatic recurrence after treatment. (**a**) Schematic of the experimental approach. Tumor-derived MDA-MB-231 cells were sorted into DsRed+ or GFP+ populations and treated with 10 nM of paclitaxel for 48 h. Fresh media were added and the cells were cultured for an additional 30 days to determine how many colonies would form. (**b**) The number of colonies that formed by day 20 of the recovery period was counted (*n* = 10); *p*-value is displayed to compare GFP versus DsRed (two-tailed unpaired *t*-test). (**c**) Schematic depicting the experimental timeline: primary tumors were surgically resected 25 days post implantation following one IV dose of neoadjuvant chemotherapy. Three days after surgery, the mice were treated with four doses of chemotherapy. Control mice were sacrificed to assess metastatic burden 3 days after treatment, whereas mice treated with paclitaxel were sacrificed 7 days later. (**d**) Whole mount fluorescent image of lung (top) and liver (bottom) of one representative mouse per treatment group. (**e**,**f**) Percentage of fluorescent (DsRed+ and GFP+) cells in (**e**) lung and (**f**) liver measured by flow cytometry analysis. The ratio of mice with detectable metastasis over the total number in the treatment group is displayed for each condition. The percentage of metastatic burden in the paclitaxel-treated group (Pax) that was sacrificed 3 days after the last treatment (day 35) presented in Figure 3 is shown as a comparison (N = 2, *n* = 13–28); treatment versus control (1-way ANOVA with Dunnett (recommended) multiple comparison test). (**g**,**h**) The percentage of DsRed+ and GFP+ cells in the lung (**g**) and liver (**h**) was quantified by flow cytometry (N = 2, *n* = 13–28); *p*-value is displayed for GFP versus DsRed cells (ordinary 2-way ANOVA with Sidak multiple comparison post-test).

Our initial findings revealed that MDA-MB-231 cells that experience hypoxia in the primary tumor were less sensitive to doxorubicin and paclitaxel both ex vivo and in vivo after metastasizing to the lung and liver. Next, we questioned whether post-hypoxic cells would be more likely to contribute to recurrence after treatment. In a preliminary assessment, we used an ex vivo approach. Tumor-derived DsRed+ or GFP+ cells were treated for 48 h with 10 nM of paclitaxel, followed by a recovery period of 30 days. GFP+ cancer cells formed 5 times more colonies than DsRed+ cells (Figure 4a,b). To test the ability of the cancer cells to recur and cause relapse in vivo, we sacrificed paclitaxel-treated mice on day 42, one week after the last dose of treatment (PAX-II), whereas the vehicle control group was sacrificed on day 35 (Figure 4c). At the time of removal, the tumors contained approximately 20% GFP+ cells (Figure S5j). The mouse body weight was monitored over the course of the experiment (Figure S5k,l). The total metastatic burden in mice treated with paclitaxel was nearly equivalent to that of the control group, suggesting that the population of micrometastatic cells that remain after treatment are sufficient to cause relapse (Figure 4d–f). A higher fraction of GFP+ cancer cells was found at both metastatic sites when compared to the control group (Figure 4g,h).

Taken together, the data demonstrate that even though paclitaxel is effective at drastically reducing metastatic burden in the lung and liver (Figure 2b and Figure 3b), cells that remain at these sites can quickly recover and proliferate once the treatment stops. Importantly, our results demonstrate that even though the primary tumor was composed of only 20% GFP+ cells at the time of tumor resection, GFP+ cells contributed 2–3 times more than DsRed+ cells to metastatic recurrence in the lung and liver.

### 3.5. Post-Hypoxic Cells Develop a Breast Cancer Stem Cell-like Phenotype

**Figure 5:**
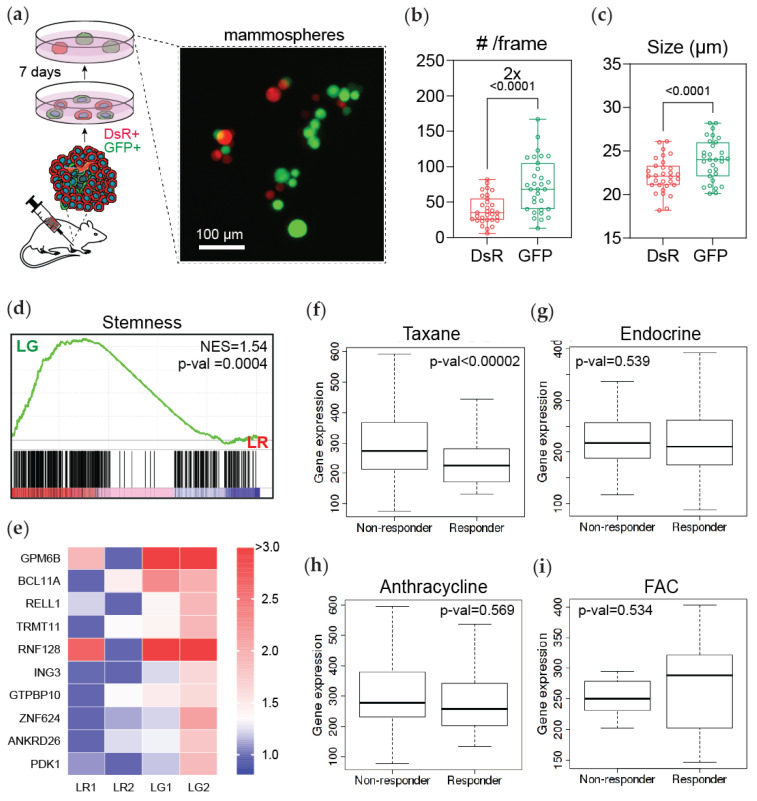
Post-hypoxic cells develop a breast cancer stem cell-like phenotype. (**a**) Tumor-derived MDA-MB-231 cells were immediately transferred to mammosphere formation media for a 7-day period and imaged by fluorescent microscopy. (**b**,**c**) The number and size of mammospheres that formed were assessed by fluorescent image analysis and normalized by the initial number of DsRed+ and GFP+ cells (N = 3, *n* = 6); *p*-value is displayed to compare GFP versus DsRed (two-tailed paired *t*-test). (**d**) A GSEA of gene set associated with stemness (Zhang et al., 2008) in DsRed+ (LR) and GFP+ (LG) cancer cells purified from lungs. Normalized enrichment score (NES) and *p*-value (*p*-val) are displayed. (**e**) Heatmap of the expression of the top 10 genes driving the enrichment of stemness in (**d**) in DsRed+ (LR1 and LR2) and GFP+ (LG1 and LG2) lung-derived cancer cells. (**f**–**i**) ROC analysis of the top 10 stemness-associated genes for patients that did or did not have a response to treatment with (**f**) taxane alone; (**g**) endocrine therapy; (**h**) anthracycline alone; or (**i**) fluorouracil, Adriamycin (doxorubicin), and Cytoxan (FAC) therapies.

Hypoxia has been implicated in chemoresistance through several HIF-1α dependent mechanisms, including upregulation of ABC transporters, DNA repair mechanisms, autophagy, enrichment in cancer stem cell-like properties, and protection from apoptosis and senescence [24]. Previous studies have demonstrated that HIF-1α is required for CSC enrichment and CSCs are more chemoresistant than cells that do not have a CSC phenotype [30,36,37]. To investigate the cancer stem cell-like potential of cells that experienced intratumoral hypoxia (GFP+), we harvested cancer cells from recently harvested tumors and cultured them in mammosphere formation media following reoxygenation (Figure 5a). After 7 days, we determined that GFP+ cells generated 2 times more mammospheres and were larger in size than DsRed cells (Figure 5b,c).

To further investigate the cancer stem cell-like phenotype in the GFP+ cells harvested from lung metastasis, we used a GSEA analysis. Interestingly, GFP+ cells were significantly enriched for a stemness gene-signature (Zhang et al., 2008 [38]) compared to DsRed+ cells (Figure 5d). The top 10 genes driving this result visibly clustered the DsRed+ versus the GFP+ cancer cells derived from the lung (Figure 5e). By utilizing the top 10 stemness genes, we performed receiver operating characteristic (ROC) analysis to determine if this signature could predict benefit from a specific treatment [28]. Our results showed that patients with breast cancer and high expression of this signature were less likely to respond to taxane therapy (Figure 5f). In contrast, the signature could not predict response to endocrine; anthracycline; or fluorouracil, Adriamycin (doxorubicin), and Cytoxan (FAC) treatment regimens (Figure 5g–i and Figure S6). Together these results suggest that hypoxia enriches the expression of genes related to stemness, which may promote the chemoresistant phenotype of GFP+ cells.

## 4. Discussion

Metastasis is the cause of 90% of solid cancer-related deaths, and this is in part due to chemoresistance. Furthermore, intratumoral hypoxia has been widely associated with chemoresistance via several mechanisms that depend on HIF-signaling, including HIF-transcriptional activation of ATP-binding cassette (ABC) transporter family genes to promote drug efflux [39,40], hypoxia-dependent autophagy via HIF-1 to promote resistance [41,42], and HIF-1-regulated decreased drug-induced senescence [43]. In our previous work, we designed and implemented a hypoxia fate-mapping system in MDA-MB-231 and 4T1 cells and determined that cells that experience intratumoral hypoxia have a 5-fold increase in their ability to form lung metastasis [25]. In the current study, we ask whether cells that experience hypoxia in the primary tumor and become reoxygenated (termed post-hypoxic cells) as they metastasize to other organs via the bloodstream are resistant to chemotherapy. Gene set enrichment analysis (GSEA) was used as an assessment tool to demonstrate that cells that experienced intratumoral hypoxia showed enrichment of previously published chemoresistance signatures. Moreover, our studies using both ex vivo and in vivo approaches revealed that post-hypoxic MDA-MB-231 cells show decreased sensitivity to paclitaxel and doxorubicin. In line with our observations, in vitro studies have demonstrated that MDA-MB-231 cells exposed to 1% O_2_ showed increased resistance to paclitaxel [44,45] and doxorubicin [43] via a HIF-1α-dependent mechanism. This suggests that the post-hypoxic resistance observed in GFP+ cells might be linked to HIF-1α signaling, which we have previously demonstrated to be enriched in GFP+ cancer cells isolated from both mouse tumors and lungs [25]. The GFP+ MDA-MB-231 cells did not show resistance to cisplatin or 5-FU. The 4T1 post-hypoxic cells were also less sensitive to paclitaxel in vitro and in vivo. One caveat of our study is that it was conducted using only MDA-MB-231 and 4T1 cell lines. Thus, our results do not rule out that in other cell lines, cancer types, or patients, hypoxia also alters sensitivity to cisplatin or 5-FU. For example, one report demonstrated hypoxia-induced cisplatin resistance in MDA-MB-231 cells in vitro [46], which was not observed in our set-up. This difference in results might suggest that resistance to cisplatin is an effect of active hypoxia as cells in the aforementioned study were exposed and treated for 24 and 48 h, whereas our study is focused on re-oxygenated cells in the lung. In another study, hypoxia-induced resistance to 5-FU was noted in gastric cancer cell lines (AGS and MKN28) [47].

HIF-1α signaling has been implicated in several mechanisms of chemoresistance, particularly enrichment in a cancer stem cell-like phenotype. BCSCs have enhanced resistance to cytotoxic agents, and the percentage of BCSCs following chemotherapy is increased compared with their before treatment status [30]. By utilizing GSEA, our results demonstrate that cells that experience intratumoral hypoxia and form distant metastasis in the lung have enrichment in hallmark hypoxia and cancer stem cell-like signaling pathways. Moreover, our preliminary assessment suggests that tumor-derived GFP+ cells have increased cancer stem cell-like properties and form more mammospheres, a well-accepted phenotype of BCSCs.

In our previous work, we also determined that cells that experience intratumoral hypoxia have a ROS-resistant phenotype that enhances survival in the bloodstream and ultimately drives metastasis. Chemotherapy can promote ROS accumulation, overwhelming redox homeostasis and leading to ROS-induced apoptosis [48]. Both paclitaxel and doxorubicin promote ROS accumulation [49,50]. Thus, it is also plausible that the ROS-resistant phenotype of the GFP+ cells may also contribute to the observed resistance.

## 5. Conclusions

In this work, we used a hypoxia fate-mapping system to investigate whether intratumoral hypoxia causes chemoresistance in murine models of breast cancer. Overall, our results show that cells that experience hypoxia in the primary tumor are (1) more equipped to metastasize, (2) less sensitive to chemotherapeutics, and (3) more likely to cause recurrence. Biomarkers are urgently needed to identify post-hypoxic cells in primary tumors and at metastatic sites in order to develop a strategy to target them and prevent recurrence.

## Data Availability

RNA sequencing data are available in the GEO database under the accession codes GSE126609 (tumor) and GSE136372 (lung).

## Supplementary Materials

The following are available online at https://www.mdpi.com/article/10.3390/cancers13215509/s1, Figure S1: Post-hypoxic tumor cells are less sensitive to chemotherapy ex vivo, Figure S2: 4T1 post-hypoxic tumor cells are resistant to Paclitaxel ex vivo, Figure S3: Ex vivo screening of post-hypoxic tumor cells predicts treatment outcome in vivo, Figure S4: GFP+ cells are enriched in signatures associated with chemoresistance, Figure S5: GFP+ cancer cells are resistant to Doxorubicin and Paclitaxel in vivo, Figure S6: GFP+ cells that metastasize retain breast cancer stem cell phenotype, Table S1: Compiled gene signature of genes associated with resistance to Paclitaxel.
