# Supplementary material for: Post-Hypoxic Cells Promote Metastatic Recurrence after Chemotherapy Treatment in TNBC
Source: Cancers (Basel). 2021 Nov 2;13(21):5509. doi: 10.3390/cancers13215509 (PMC8583122; doi:10.3390/cancers13215509)
Supplement: Supplementary file 1 [file cancers-13-05509-s001.zip › cancers-1412062-supplementary.pdf]

cancers

MDPI

Supplementary Materials: Post-hypoxic Cells Promote Metastatic Recurrence after Chemotherapy Treatment in TNBC

Inês Godet, Mahelet Mamo, Andrea Thurnheer, D. Marc Rosen and Daniele M. Gilkes

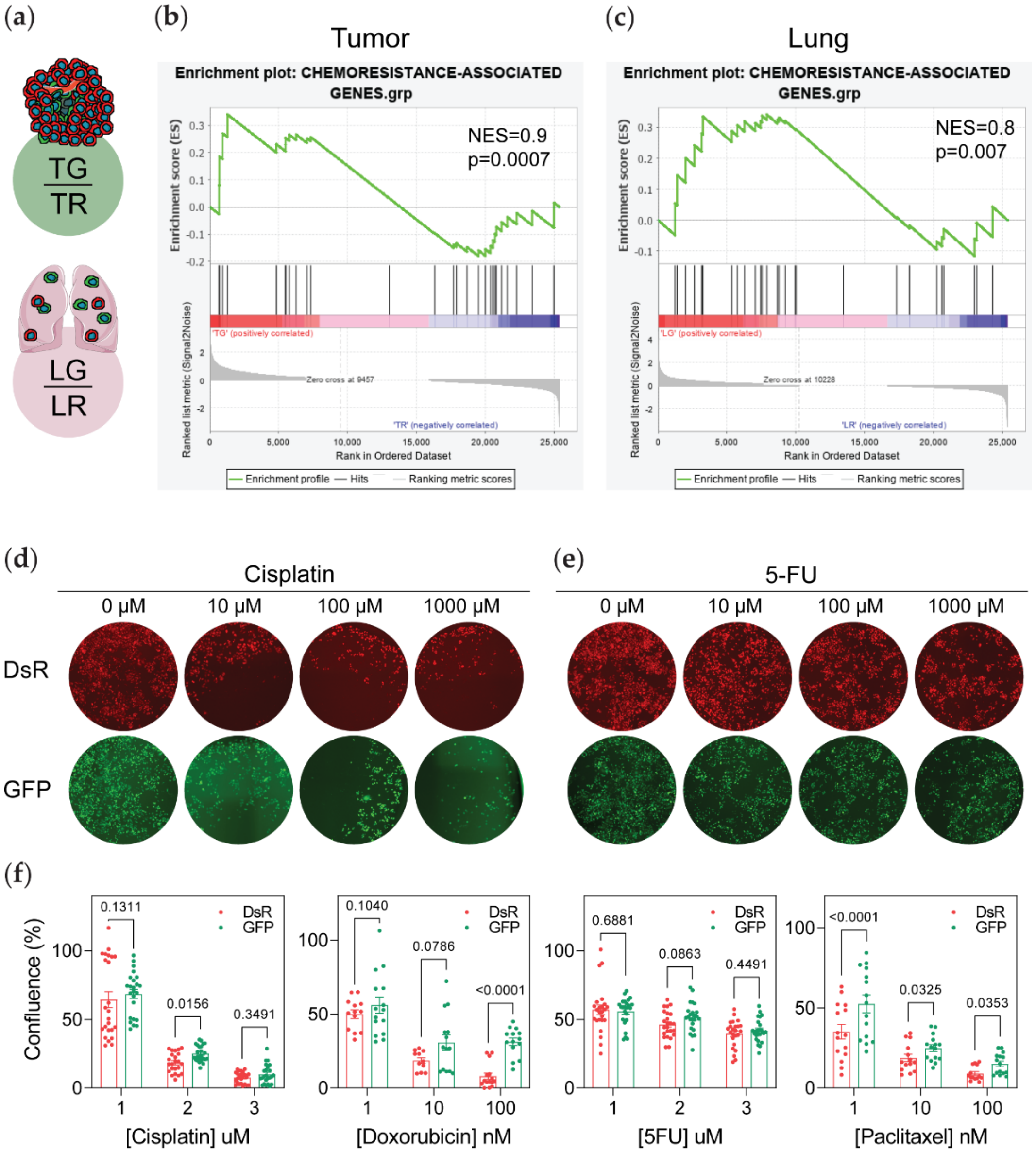

Figure S1. Post-hypoxic tumor cells are less sensitive to chemotherapy ex vivo. (**a**) Schematic of RNA sequencing set-up comparing GFP+ (TG) versus DsRed+ (TR) tumor cells and GFP+ (LG) versus DsRed+ (LR) metastatic cells in the lung as previously reported [23]. (**b–c**) Gene Set Enrichment Analysis (GSEA) of gene sets associated with chemoresistance of DsRed+ and GFP+ cancer cells purified from tumors (**b**) and lungs (**c**). Normalized enrichment score (NES) and *p*-value (*p*-val) are displayed. (**d–e**) Fluorescent images of tumor-derived DsRed+ and GFP+ sorted cells treated with Cisplatin (**d**) and 5FU (**e**) from IC_{50} analysis in Figure 1c,e. (**f**) Quantification of % area of sorted DsRed+ or GFP+ cells treated as indicated in (**d**), (**e**) and Figure 1g–h (N = 3, *n* = 3); *p*-value is displayed to compare GFP versus DsRed via 2-way ANOVA with Sidak multiple comparison post-test.

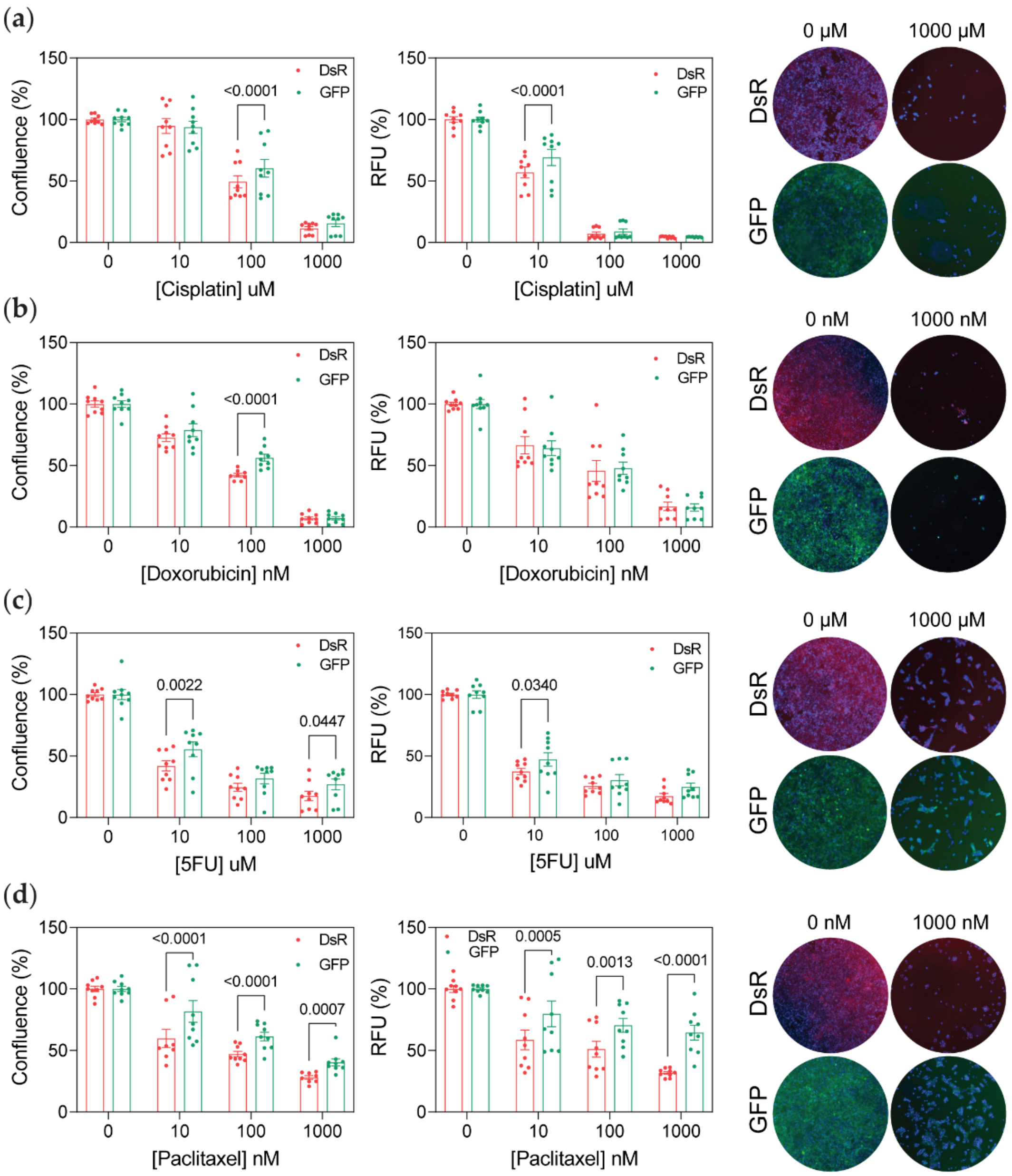

Figure S2. 4T1 post-hypoxic tumor cells are resistant to Paclitaxel ex vivo. (**a**–**d**) Tumors that formed from the orthotopic injection of 4T1 hypoxia fate-mapping cells were sorted into DsRed+ or GFP+ populations and then cultured in vitro to determine the IC_{50} of Cisplatin (**a**), Doxorubicin (**b**), 5-Fluorouracil (**c**) or Paclitaxel (**d**) for 48 h. Cell viability is displayed using the area of the cell culture well covered by nuclear DAPI staining (left) or using a Presto Blue assay (middle) (N = 1, *n* = 3); *p*-value is displayed to compare GFP versus DsRed via 2-way ANOVA with Sidak multiple comparison post-test. RFU = Relative fluorescence units. Fluorescent images of tumor-derived DsRed+ and GFP+ sorted cells treated highest dose of each chemotherapy (left).

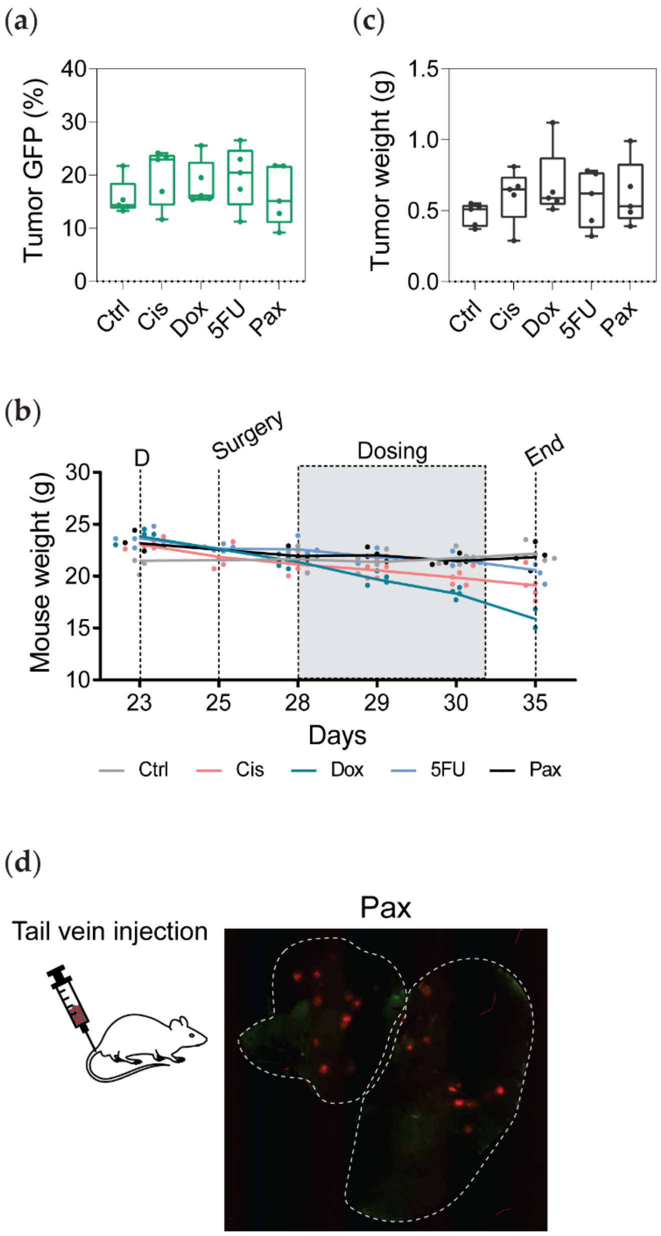

Figure S3. Ex vivo screening of post-hypoxic tumor cells predicts treatment outcome in vivo. (**a**) Percentage of GFP+ cells derived from resected tumors measured by flow cytometry. (**b**) Mouse weight during treatment. (**c**) Tumor weights after surgical resection. (**d**) Fluorescent whole mount image lungs of NSG mice injected with hypoxia fate-mapping MDA-MB-231 cells via tail vein and treated with 5 doses of Paclitaxel treatment.

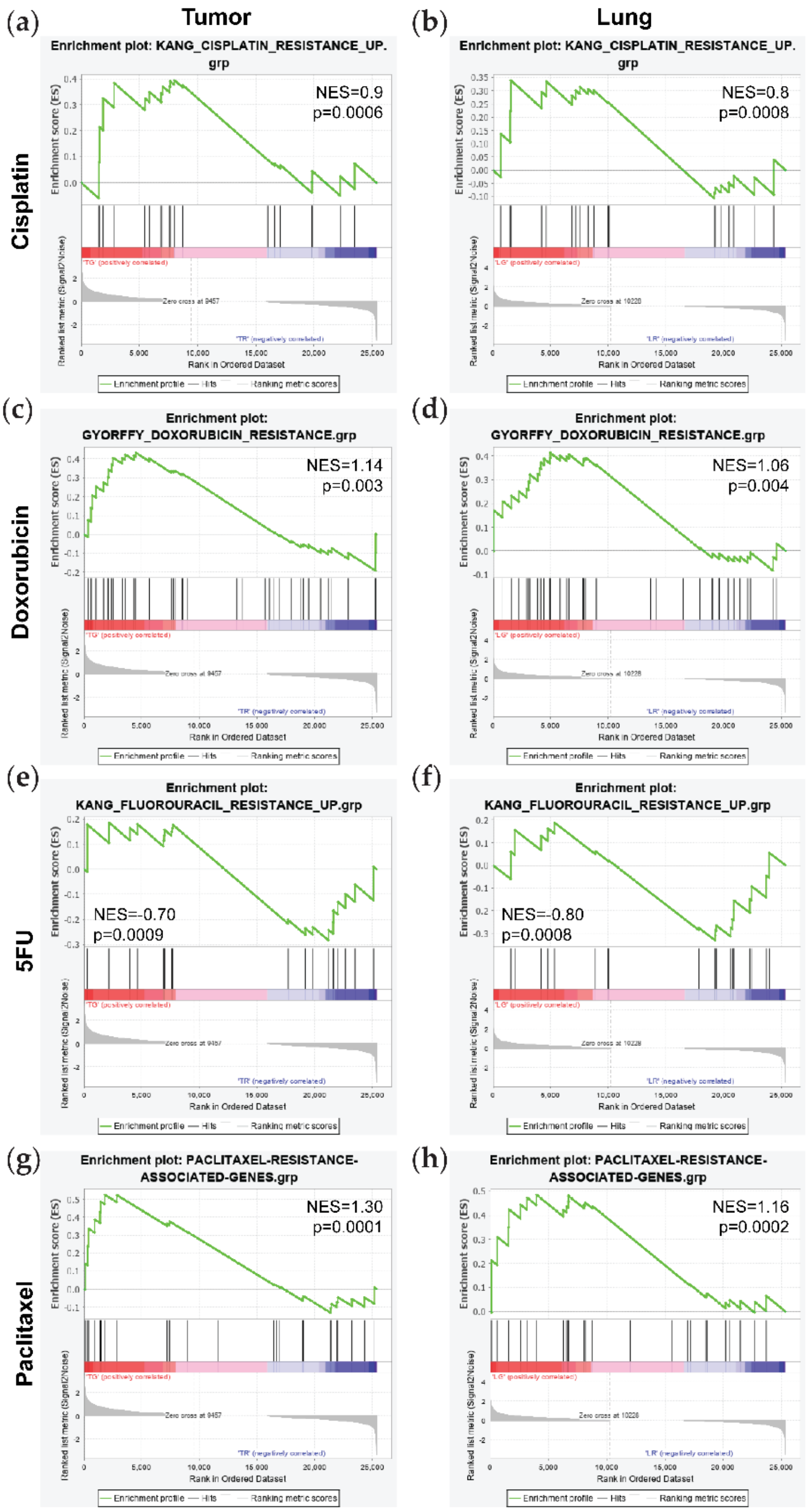

Figure S4. GFP+ cells are enriched in signatures associated with chemoresistance. (**a**–**h**) GSEA of gene signatures associated with resistance to Cisplatin (**a**–**b**), Doxorubicin (**c**–**d**), 5-FU (**e**–**f**), or Paclitaxel in GFP+ (TG) compared to DsRed+ (TR) cells after sorting directly from the tumors (**a**,**c**,**e**,**g**) or lungs (**b**,**d**,**f**,**h**). Normalized enrichment score (NES) and *p*-value (*p*-val) are displayed.

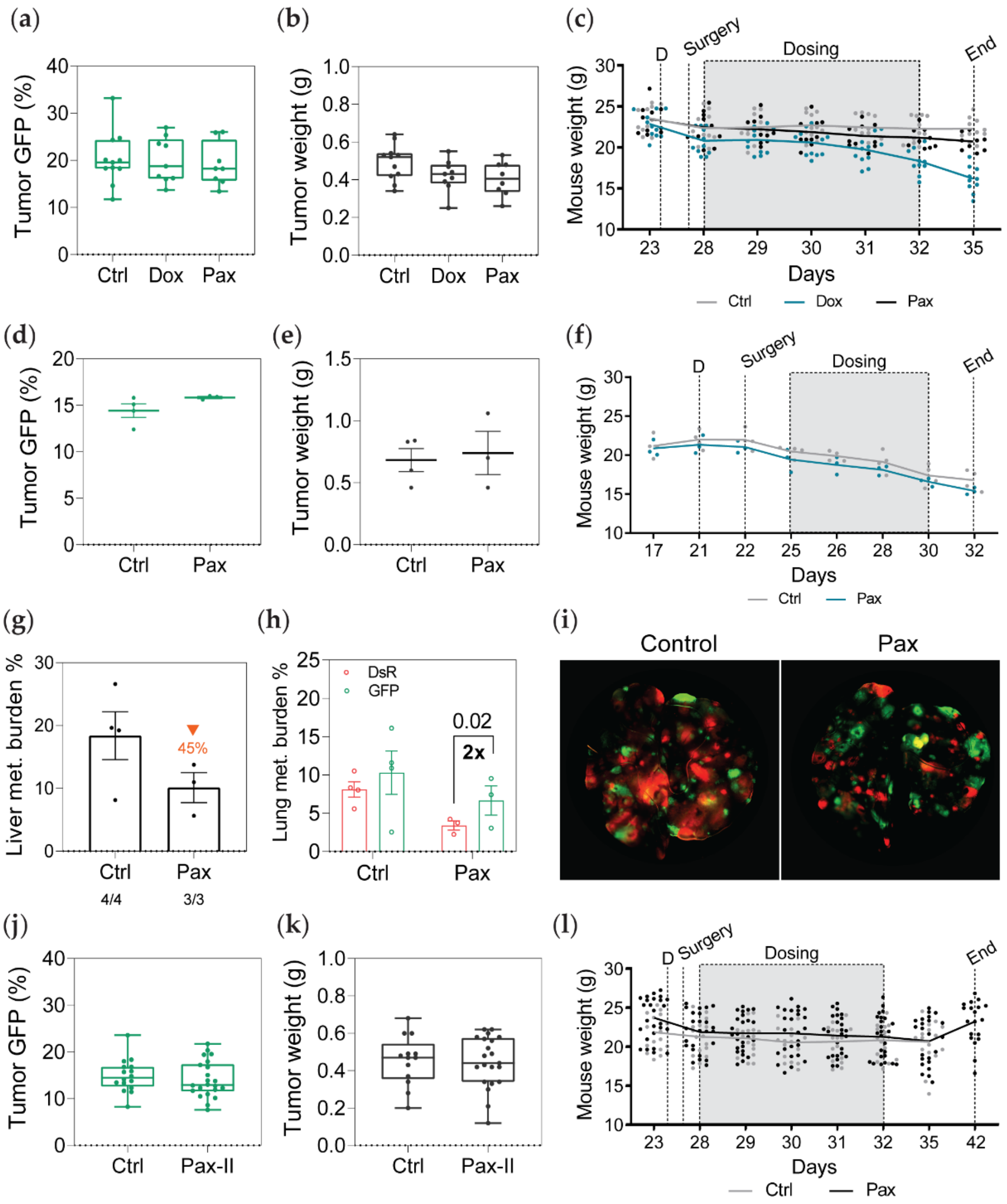

Figure S5. GFP+ cancer cells are resistant to Doxorubicin and Paclitaxel in vivo. (**a**,**b**) Percentage of GFP+ cells (**a**) and weights (**b**) of resected MDA-MB-231 tumors. (**c**) Weights of NSG mice orthotopically injected with hypoxia fate-mapping MDA-MB-231 cells during experimental set-up. (**d**,**e**) Percentage of GFP+ cells (**d**) and weights (**e**) of resected 4T1 tumors. (**f**) Weights of NSG mice orthotopically injected with hypoxia fate-mapping 4T1 cells during experimental set-up. (**g**) Percentage of fluorescent (DsRed+ and GFP+) cells measured by flow cytometry analysis (orange arrow indicates decreased metastatic burden). The ratio of mice with detectable metastasis over the total number in the treatment group is displayed for each condition. (**h**) The percentage of DsRed+ or GFP+ cells in the lung as measured by flow cytometry (n = 3–4); p-value is displayed to compare GFP versus DsRed (one-tailed paired t-test). (**i**) Whole mount fluorescent image of lungs from one representative mouse per treatment group. (**j**–**k**) Percentage of GFP+ cells (**j**) and weight (**k**) of resected MDA-MB-231 tumors and quantified by flow cytometry. (**l**) Weights of NSG mice orthotopically injected with hypoxia fate-mapping MDA-MB-231 cells during experimental set-up where Paclitaxel-treated mice were sacrificed on day 42.

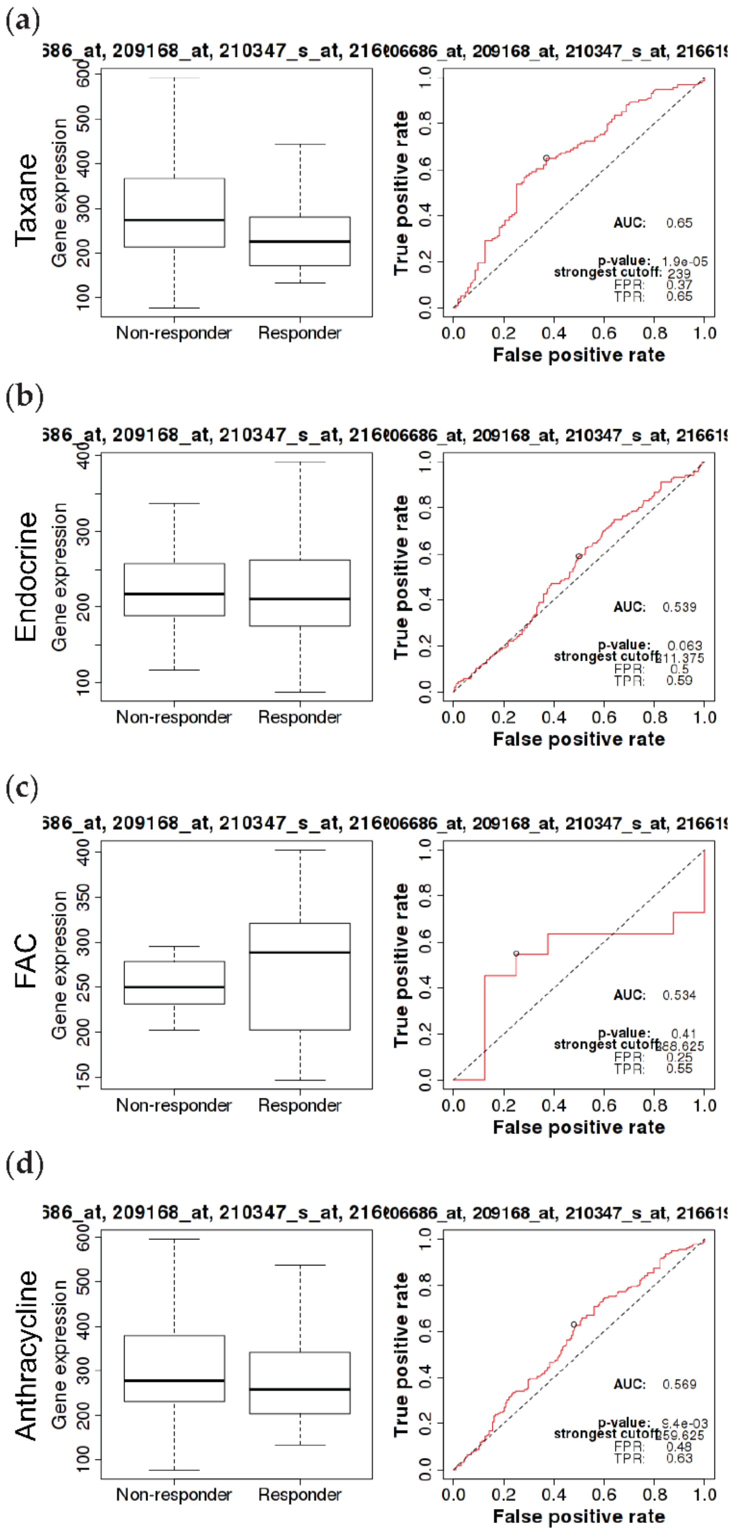

Figure S6. GFP+ cells that metastasize retain breast cancer stem cell phenotype. (**a**–**d**) ROC analysis using the top-10 stemness associated-genes in patients treated with (**a**) Taxane, (**b**) Endocrine, (**c**) Antracycline and (**d**) FAC therapies.

Table S1. Compiled gene signature of genes associated with resistance to Paclitaxel.

Gene	Reference
IL6	Jurj A, Pop LA, Zanoaga O, Ciocan-Cârtiță CA, Cojocneanu R, Moldovan C, Raduly L, Pop-Bica C, Trif M, Irimie A, Berindan-Neagoe I, Braicu C. New Insights in Gene Expression Alteration as Effect of Paclitaxel Drug Resistance in Triple Negative Breast Cancer Cells. Cell Physiol Biochem. 2020 Jul 4;54(4):648-664. doi: 10.33594/000000246. PMID: 32619350.
CXCL8	
VEGFA	
EGR1	
PTGS2	
TRIB1	Murakami H, Ito S, Tanaka H, Kondo E, Kodera Y, Nakanishi H. Establishment of new intraperitoneal paclitaxel-resistant gastric cancer cell lines and comprehensive gene expression analysis. Anticancer Res. 2013 Oct;33(10):4299-307. PMID: 24122996.
KIF23	
EBB2IP	
ATAD2	
PHF19	
GBP1	Duan Z, Lamendola DE, Duan Y, Yusuf RZ, Seiden MV. Description of paclitaxel resistance-associated genes in ovarian and breast cancer cell lines. Cancer Chemother Pharmacol. 2005 Mar;55(3):277-85. doi: 10.1007/s00280-004-0878-y. Epub 2004 Nov 24. PMID: 15565326.
TLR6	
CATP3	
TSBP	
MDR1	
TMEM243	
BCAP29	
NFKB2	
ABCC10	Dorman SN, Baranova K, Knoll JH, Urquhart BL, Mariani G, Carcangiu ML, Rogan PK. Genomic signatures for paclitaxel and gemcitabine resistance in breast cancer derived by machine learning. Mol Oncol. 2016 Jan;10(1):85-100. doi: 10.1016/j.molonc.2015.07.006. Epub 2015 Aug 22. PMID: 26372358; PMCID: PMC5528934.
BCL2	
BCL2L1	
BIRC5	
BMF	
FGF2	
FN1	
MAP4	
MAPT	
NFKB2	
SLCO1B3	
TWIST1	
CSAG2	
